# Supplementary material for: Application of simultaneous uncertainty quantification for image segmentation with probabilistic deep learning: Performance benchmarking of oropharyngeal cancer target delineation as a use-case
Source: medRxiv. 2023 Feb 24:2023.02.20.23286188. Preprint. [Version 1] doi: 10.1101/2023.02.20.23286188 (PMC9980236; doi:10.1101/2023.02.20.23286188)
Supplement: 1 [file NIHPP2023.02.20.23286188v1-supplement-1.pdf]

medRxiv preprint doi: https://doi.org/10.1101/2023.02.20.23286188; this version posted February 24, 2023. The copyright holder for this preprint (which was not certified by peer review) is the author/funder, who has granted medRxiv a license to display the preprint in perpetuity.
It is made available under a CC-BY 4.0 International license .

# Appendix A: Supplementary Methods

Additional details on the MDA external validation dataset are provided here. For the 67 patients included, FDG-PET/CT scans were acquired from various GE Medical Systems scanners. Specifically, Discovery RX (n=27), Discovery STE (n=26), Discovery ST (n=12) and Discovery HR (n=2) models were used. Image acquisition parameters are shown in **Table A1**. A 90-minute uptake period of rest was used for all patients. Attenuation corrected images were reconstructed using an ordered subset expectation maximization (OSEM) iterative algorithm (2 iterations, 18-24 subsets, 5mm Gaussian filter).

| **Acquisition Parameter** | **CT** | **PET** |
|---|---|---|
| In-plane resolution (mm) | 0.98 | 5.47 |
| Slice thickness (mm) | 3.75 | 3.27 |
| Exposure time (ms)* | 566 (500-676) | NA |
| X-ray tube current (mA)* | 200 (100-296) | NA |
| KVP (kV)* | 100 (100-120) | NA |
| Dose (Mbq)** | NA | 375 (281-729) |

**Table A1:** PET/CT image acquisition parameters. All values were the same across all patients unless a parenthesis is shown, where the median and range are displayed. * only apply to CT data; ** only apply to PET data.

medRxiv preprint doi: https://doi.org/10.1101/2023.02.20.23286188; this version posted February 24, 2023. The copyright holder for this preprint (which was not certified by peer review) is the author/funder, who has granted medRxiv a license to display the preprint in perpetuity.
It is made available under a CC-BY 4.0 International license .

# Appendix B: Additional Qualitative Analysis

In this section, we present qualitative results for select cases in the MDA holdout dataset. Specifically, we describe our interpretations of model predictions and uncertainty maps relative to ground truth across multiple axial image slices, similar to how a case would be reviewed in the clinic. For simplicity, we only describe results of the MD Dropout Ensemble model. "High" and "low" values are relative to median values described in the main text (e.g., high DSC is greater than 0.61).

*Case 1: Low performance, high certainty.*

Here we describe a case with low DSC (0.43) but high certainty ($-U_P = -0.43$). Axial slice representations from superior to inferior slices for this case are shown in **Figure B1**. As can be seen in the inferior slices (slice 54), there is initially a relatively large degree of uncertainty about the beginning of the prediction. Subsequently (slice 66), the model correctly predicts the tumor at the left base of tongue, with a simultaneous region of uncertainty appearing at the right base of tongue, likely secondary to the high PET signal causing a potential area of false positivity. This false positive PET signal is not ultimately included in the predicted segmentation mask, which in this case is seen as a desired outcome. More superiorly (slice 79), in terms of uncertainty and the resultant prediction, the model seems to have erroneously localized to the hyper-metabolic core of the primary tumor. Finally, at the most superior slices (slice 90), it is noted that there was metal streak artifact induced by dental hardware, which may have interfered with model inference and subsequent uncertainty quantification, as no prediction was generated. Main takeaways from this case include the model overemphasizing PET signal (which has been previously noted in PET/CT auto-segmentation models) which is also reflected in the resultant uncertainty measures. Moreover, the image artifact may also impact performance and uncertainty estimation.

medRxiv preprint doi: https://doi.org/10.1101/2023.02.20.23286188; this version posted February 24, 2023. The copyright holder for this preprint (which was not certified by peer review) is the author/funder, who has granted medRxiv a license to display the preprint in perpetuity.
It is made available under a CC-BY 4.0 International license .

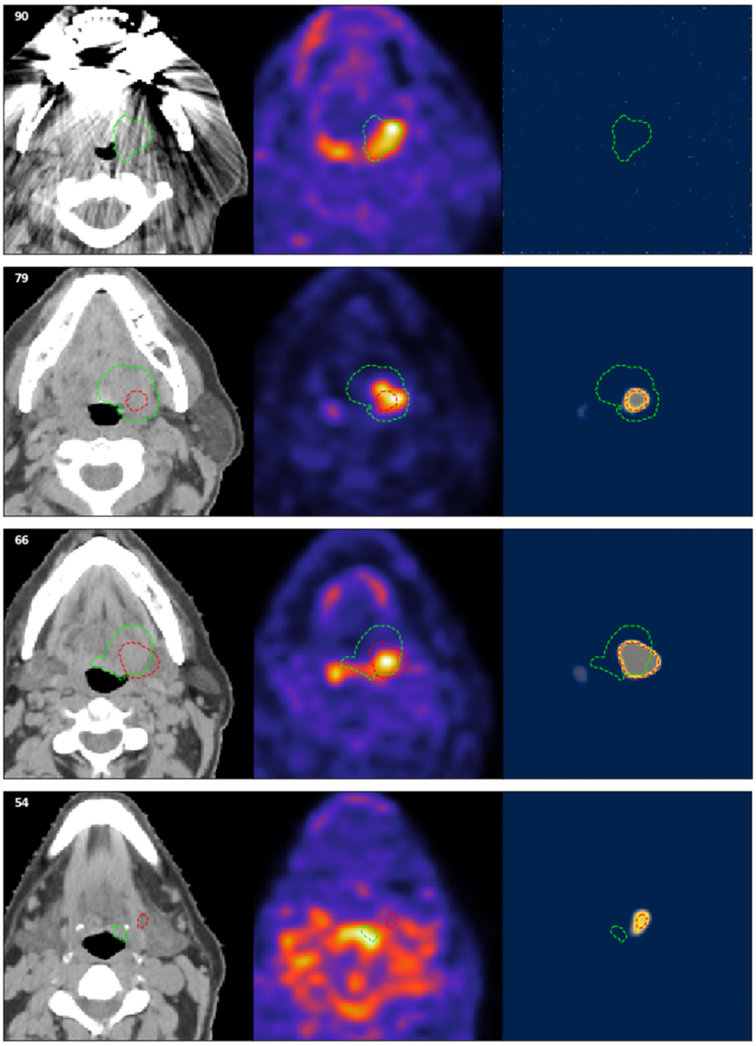


**Figure B1**: Additional qualitative investigation of a case with low performance and high certainty. Number in top left corner = slice number; green dotted outline = ground-truth segmentation, red dotted outline = predicted segmentation. Blue, gray, and yellow colors in uncertainty maps correspond to low, medium, and high model uncertainty, respectively.

*Case 2: High performance, low certainty.*

Here we describe a case with high DSC (0.64) but low certainty ($-U_P = -0.5$). Axial slice representations from superior to inferior slices for this case are shown in **Figure B2**. In the inferior-most slices (slices 18-27), uncertainty is noted near the larynx, likely a byproduct of high PET signal. As before, this false positive PET signal is not ultimately included in the predicted segmentation mask, which in this case is seen as a desired outcome. More superiorly (slice 61), the model begins to predict a segmentation on only the right side of the base of tongue, when in reality the ground-truth is a bilateral segmentation. Importantly, the model starts to note uncertainty on the contralateral part of the image, which is a desired outcome. As we move further superiorly towards the tonsils (slice 70) the tumor begins to exhibit an uncommon presentation (discontinuous fragment, bilateral in both tonsils), but the prediction better starts to approximate the ground-truth; the uncertainty previously demonstrated at the contralateral side (left) is still present but has now started to become included in the predicted segmentation. Continuing superiorly (slice 78), there is still high

medRxiv preprint doi: https://doi.org/10.1101/2023.02.20.23286188; this version posted February 24, 2023. The copyright holder for this preprint (which was not certified by peer review) is the author/funder, who has granted medRxiv a license to display the preprint in perpetuity.
It is made available under a CC-BY 4.0 International license .

uncertainty in the discontinuous fragment but the model is able to generate a reasonable prediction, however the model eventually starts to generate an implausible prediction in an air space (slice 85) as the prediction begins to generate a bilateral segmentation erroneously. As with the previous case, towards the superior-most part of the image (slices 90-94), metal streak artifact induced by dental hardware may alter the predictions and uncertainty estimation; notably, the prediction ignores the false positive PET signal. Main takeaways from this case include uncommon tumor presentations (e.g., fragmentation of tumor from one continuous piece to two pieces) may present issues in generating prediction and uncertainty. Moreover, as before, image artifacts may impact predictions and uncertainty estimation.

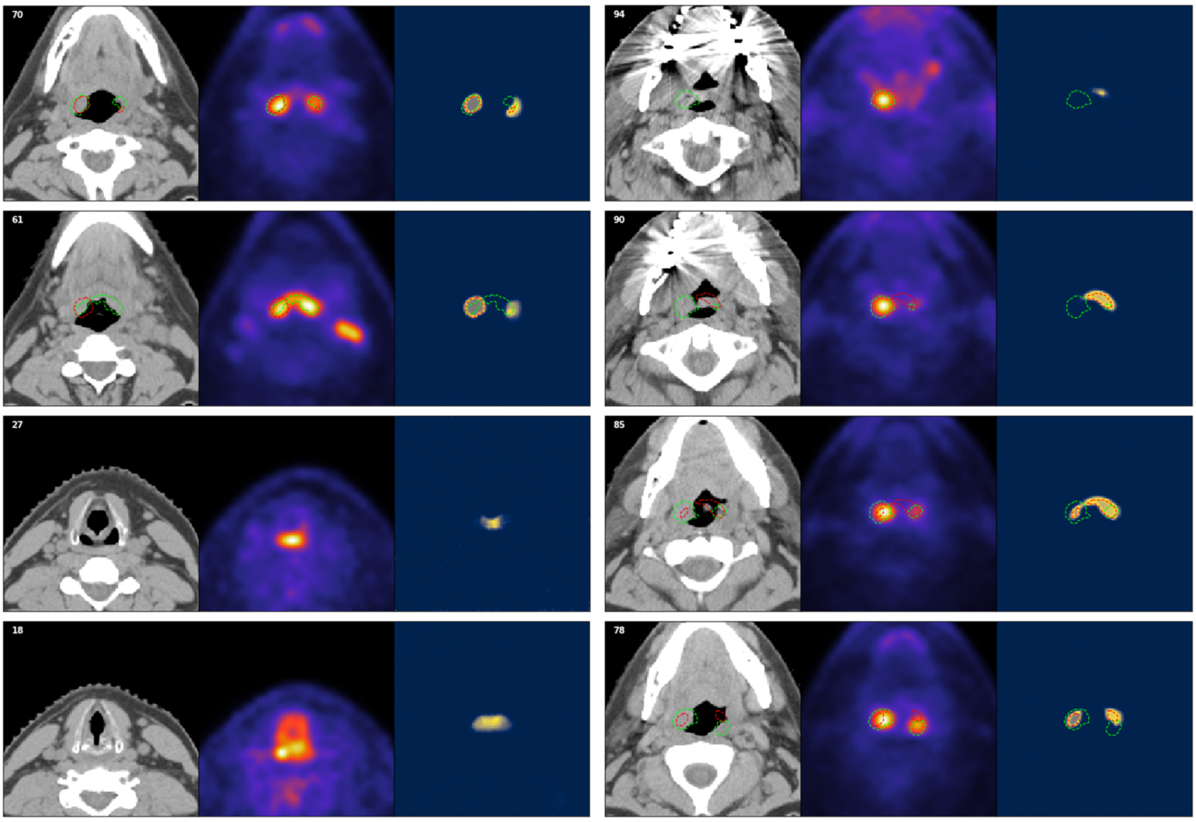


**Figure B2**: Additional qualitative investigation of a case with high performance and low certainty. Number in top left corner = slice number; green dotted outline = ground-truth segmentation, red dotted outline = predicted segmentation. Blue, gray, and yellow colors in uncertainty maps correspond to low, medium, and high model uncertainty, respectively.

*Case 3: Contralateral uncertainty.*

Here we describe an interesting case with high DSC (0.64) and high certainty ($-U_P = -0.41$). Axial slice representations from superior to inferior slices for this case are shown in **Figure B3**. At the inferior slice (slice 60), the model generates the prediction correctly at the left tonsil but starts to note uncertainty at the contralateral tonsil. Subsequently, at the more superior slice (slice 70) the contralateral portion is revealed as part of the ground truth segmentation. The model is still uncertain about the area and ultimately does not include it

medRxiv preprint doi: https://doi.org/10.1101/2023.02.20.23286188; this version posted February 24, 2023. The copyright holder for this preprint (which was not certified by peer review) is the author/funder, who has granted medRxiv a license to display the preprint in perpetuity.
It is made available under a CC-BY 4.0 International license .

as part of the prediction. In other words, the contralateral uncertainty indicates a false negative area that the model is uncertain about. In a clinical workflow this would correspond to an area the clinician could choose to further investigate.

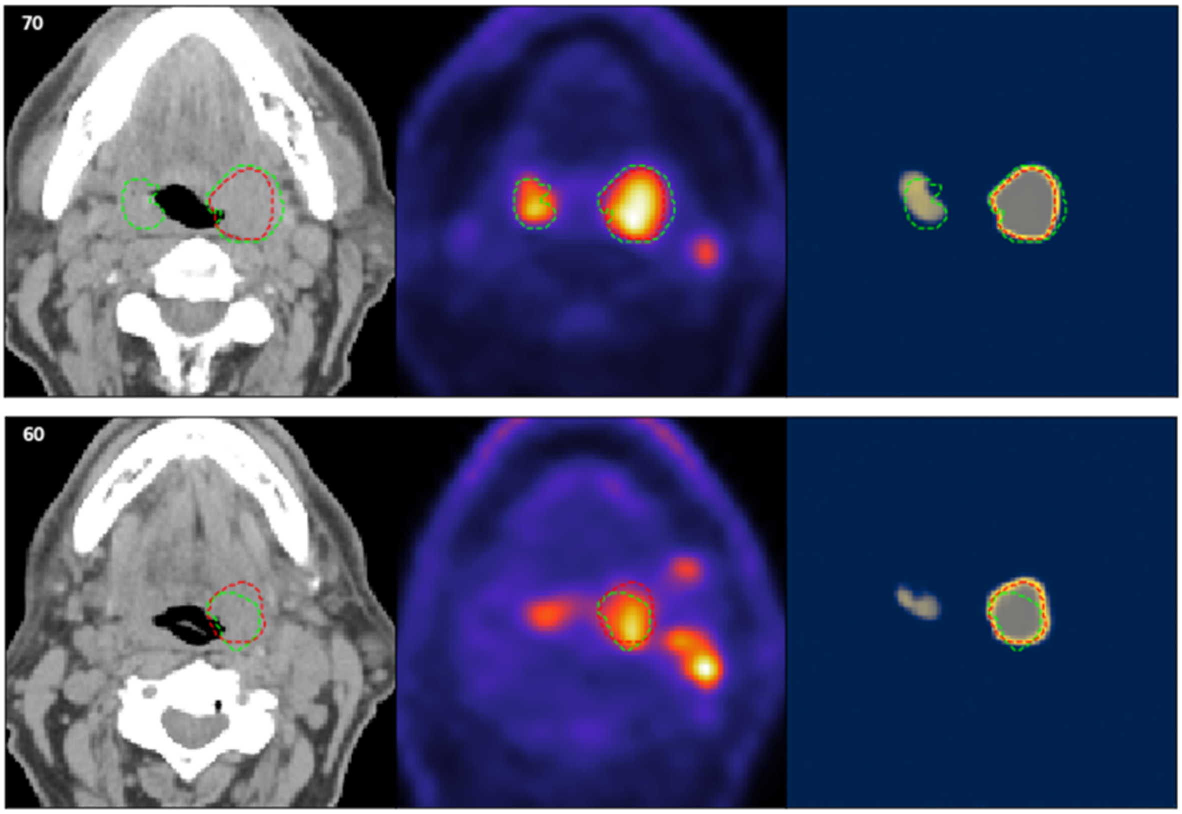


**Figure B3**: Additional qualitative investigation of a case with contralateral uncertainty. Number in top left corner = slice number; green dotted outline = ground-truth segmentation, red dotted outline = predicted segmentation. Blue, gray, and yellow colors in uncertainty maps correspond to low, medium, and high model uncertainty, respectively.

*Case 4: Nodal uncertainty.*

Here we describe an interesting case with high DSC (0.71) and high certainty ($-U_P = -0.44$). Axial slice representations from superior to inferior slices for this case are shown in **Figure B4**. In the inferior-most slice (slice 22) there is noted uncertainty in the area of high PET signal (likely spurious signal), which is not included in the prediction, which in this case is seen as a desired outcome. More superiorly (slices 61-70) a metastatic lymph node is present on the right side of the image; there is corresponding noted uncertainty about this area and it is ultimately not included in the prediction. The model is able to generate a prediction for the right base of tongue tumor without issues. Notably, as observed through the majority of other cases, metastatic lymph nodes are normally not considered by the model at all, likely due to the often large geometric distances between the nodal metastases and the primary tumors. In this case the node exhibits features (i.e. high PET signal) in close proximity to the primary tumor, which could have led to the model uncertainty about this prediction.

medRxiv preprint doi: https://doi.org/10.1101/2023.02.20.23286188; this version posted February 24, 2023. The copyright holder for this preprint (which was not certified by peer review) is the author/funder, who has granted medRxiv a license to display the preprint in perpetuity.
It is made available under a CC-BY 4.0 International license .

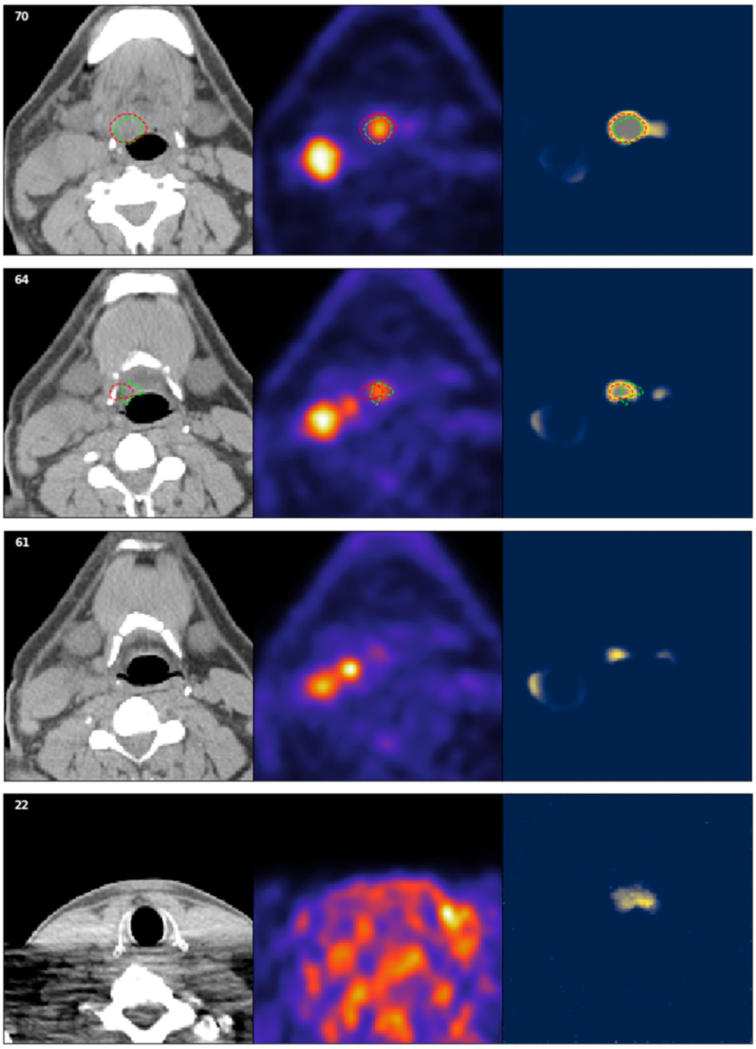


**Figure B4**: Additional qualitative investigation of a case with nodal uncertainty. Number in top left corner = slice number; green dotted outline = ground-truth segmentation, red dotted outline = predicted segmentation. Blue, gray, and yellow colors in uncertainty maps correspond to low, medium, and high model uncertainty, respectively.
